# Spatial Accessibility to Primary Care in Metropolitan France: Results Using the SCALE Spatial Accessibility Index for All Regions

**DOI:** 10.3390/ijerph21030276

**Published:** 2024-02-28

**Authors:** Ludivine Launay, Fabien Guillot, Mohand Medjkane, Guy Launoy, Olivier Dejardin

**Affiliations:** 1 Plateforme MapInMed, US PLATON, University of Caen Normandy, 14033 Caen, France; guy.launoy@unicaen.fr (G.L.); olivier.dejardin@unicaen.fr (O.D.); 2 U1086 INSERM UCN Anticipe, “Equipe Labellisée Ligue Contre le Cancer”, University Hospital of Caen, 14033 Caen, France; 3 Centre François Baclesse, 14076 Caen, France; 4 Department of Geography and Territorial Planning, University of Caen Nomandy, UMR CNRS 6266 IDEES-Caen, 14032 Caen, France; fabien.guillot@unicaen.fr (F.G.); mohand.medjkane@unicaen.fr (M.M.); 5 Department of Research, University Hospital of Caen, 14033 Caen, France

**Keywords:** spatial accessibility, health care, availability, population, spatial unit

## Abstract

Accessibility to care is a major public health issue. Various tools to assess it are available, but they do not solve the problem of scale. Moreover, accessibility is a multidimensional concept that is not taken into account with current tools. The SCALE index aims to overcome these two limitations by proposing a synthetic measure on a more precise scale than the administrative unit or the sub-municipal scale. Under the assumption of access to care facilities for all and access to the nearest facilities, the potential accessibility distance was calculated for each couple (residential area, accessible facilities). This was defined as the average distance by road that the population has to travel to access care. To take the availability of resources into account, these distances were weighted by the theoretical pressure on the facilities. The SCALE index was then calculated using a linear combination of the distances of potential accessibility to care facilities It highlights differences in accessibility at the national and regional scale. Using this index, it was possible to provide maps for all French regions and the major cities in a story-map. The major conurbation around Paris and the main urban centers has high accessibility. Low accessibility forms a “Y” shape. In conclusion, the SCALE index measures accessibility at the scale of a small geographic unit taking the proximity and the availability of health professionals into account. It is also possible to take into account the diversity of accessibility in a given territory.

## 1. Introduction

Accessibility to care is a major public health issue that is influenced by both contextual factors (health care system, care delivery, and macroeconomic environment) and individual ones (people’s knowledge about health, their beliefs, and their systems of representation) [1]. A growing body of evidence highlights a decrease in the use of health care services as travel time increases. This distance decay association has been observed in numerous studies and various contexts [2] and is now widely accepted in medical geography. 

Historically, medical density and the distance to the nearest professional have been widely used to assess health care accessibility. However, these variables suffer from important limitations. Concerning medical density, the impossibility of crossing borders and the artificial homogeneity over spatial units mean that the measurement of access to health care may be unrealistic. Concerning the distance to the nearest professional, the absence of a relationship with availability is also an important bias. To overcome these limitations, spatial accessibility indices have been developed, based mainly on the combination of accessibility (distance to facilities) and resource availability [2,3].

Gravity models figure in this family of indicators [4,5,6] and all models derived from them [7,8,9,10,11,12,13,14,15,16,17,18,19,20,21,22,23]. The most widely used version of these models is the Two-Step Floating Catchment Area model, 2SFCA [19]. The 2SFCA has three main limitations. First, the notion of availability is related only to the number of professionals in the spatial unit; yet, their availability is very different depending on their type of practice (full-time or part-time). Second, demand for care is influenced by population structure in terms of age, gender, and health status. Finally, accessibility is the same regardless of the location of the offer. A professional located close to the applicant will count as much as a professional located at the border of the area. However, it has been shown that the demand for care decreases with distance [20]. Moreover, to our knowledge, the method has not been applied to metropolitan France.

All in all, these indices are mainly focused on accessibility to a single health professional. Our hypothesis is that accessibility should be conceived as a combination of accessibility to different health resources.

The index of localized potential accessibility (LPA) calculated at the municipal level for Metropolitan France has partially compensated for the above-mentioned limitations without being intended to exceed the constraint of the scale [21]. Our hypothesis is that a potential accessibility index should be constructed in a multiscalar way to account for the complexity of socio-spatial organization. In addition, an index based on distance can overcome the effects of thresholds, breaks, and administrative limits, while including the cost of traveling the distance. The SCALE index (Spatial aCcessibility multiscALar) aims to meet these objectives [24]. Some authors have already used this index to evaluate the link between accessibility and outcomes following bariatric surgery [25] or the likelihood of receiving restorative rectal cancer surgery [26].

This study, therefore, explores spatial accessibility to care in the 11 French regions using the SCALE accessibility index [24], taking the road network into account.

## 2. Materials and Methods

### 2.1. Construction of SCALE

The SCALE index defines health accessibility as the distance (between residential area and primary health care) that the population has to travel to access care facilities. Its methodology has been previously published [24]. However, a recent improvement is to use travel time to the nearest facilities instead of straight-line distance to the nearest facilities. 

The population was located in the centroid of residential areas. Residential areas (nearly 2.8 million) were obtained by aggregating the residential areas built with BD TOPO V2.1 provided by IGN and ESRI France [24].

Health care facilities were extracted from the Base Permanente des Equipements de 2013 in a geolocalized version provided by INSEE. Primary health care facilities were included (general practitioners, physiotherapists, nurses, dentists, and pharmacists for facilities classified as primary facilities by INSEE; medical obstetricians and gynecologists, maternity wards, pediatric specialists, ophthalmologists, short-stay care facilities, and accident and emergency departments for those classified as tertiary facilities by INSEE) (n = 264,416). 

**Figure 1:**
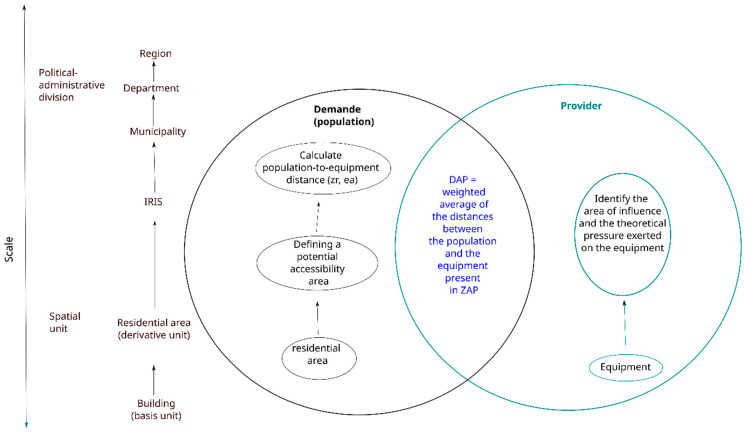
Construction of value per category.

The SCALE index was calculated in five steps and takes into account demand and provider (Figure 1).

First, a distance that ensures access to facilities for all residential areas was defined. This distance delimits an access area (the potential accessibility area = ZAP), which defines facilities accessible for the population included in calculating the potential accessibility distance. The potential accessibility distance is defined as the mean weighted distance (using distance in accordance with the travel time selected). The weight applied to the distance between each couple (residential area, facilities) takes the availability of the facilities into account. This theoretical availability is defined as the pressure applied on the facilities (as previously published). Potential accessibility distances are then transformed to obtain a reduced centered normal distribution, which is finally combined to obtain the SCALE index.

### 2.2. SCALE Index Using Road Network

Since its creation, the greatest Euclidean distance to the nearest facility (1) to delimit the potential accessibility zone for residential areas of the same IRIS (Ilots Regroupés pour l’Information Statistique, which is the smallest French administrative area for which census data are available) has been replaced by the longest travel time to drive to the nearest facility (using travel time as the impedance parameter in the calculation). The latter was calculated using Navstreets v14.0 data provided by HERE & ESRI France and using the Network Analyst extension.

Due to the performance required for this type of processing (mainly in terms of memory [27], the residential areas were processed by region. The facilities considered were those located in the region and in neighboring departments for facilities in the proximity range or those in neighboring regions for facilities in the tertiary range.

### 2.3. Modification of Geographical Unit

As SCALE is a multiscalar index, it was calculated for different geographical units. The aggregation criteria were the following: the minimum value for all residential areas in the same unit, the maximum value, mean and weighted mean by population of the residential area. All these versions were categorized according to the decile. 

All analyses were conducted using Python, ArcGIS^®^ Pro 2.5, SAS^®^ 9.4. The Origin-Destination Matrices between residential areas and facilities were calculated with a Python script developed by ESRI and adapted to fit this study. All maps were created with the RGF93 system and Lambert93 projection.

## 3. Results

The SCALE index for Metropolitan France is presented in a story map that is freely available at the following URL: L’accessibilité spatiale aux services de soins en France (arcgis.com accessed on 10 November 2023).

**Figure 2:**
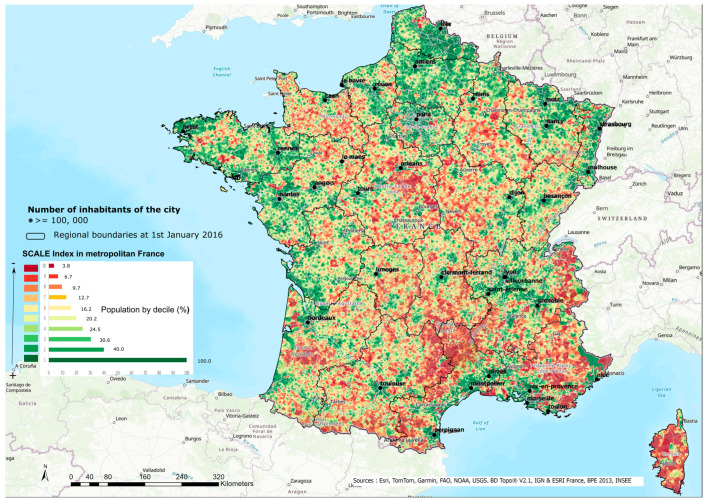
Accessibility to health care in Metropolitan France.

There are many different situations in Metropolitan France in terms of health accessibility (Figure 2). As expected, the major conurbation around Paris and the main urban centers has high accessibility (in green). On the other hand, several areas suffer from poor accessibility (in red), so accessibility is asymmetric. Among the areas with poor accessibility, there is a first axis starting from Cotentin in Normandy in the west towards the center of the Loire Valley on a line south of Orléans to Nevers in the east. This first axis joins a second axis, which extends from Champagne around Chalons-en-Champagne, descending towards the Massif Central as far as the Cévennes. These first two areas of low accessibility form a ‘Y’ shape. A second area covers the Alps from the border with Switzerland to the Mediterranean. Residential areas with the worst accessibility (the last three deciles) account for around 10% of the population. These areas of low accessibility can partly be explained by geographic and topographic factors and land use (forest areas, mountain areas, road, rail and industrial areas, etc.). However, even at this scale, the index highlights considerable differences and inequalities in access to health facilities for the populations of the areas concerned.

**Figure 3:**
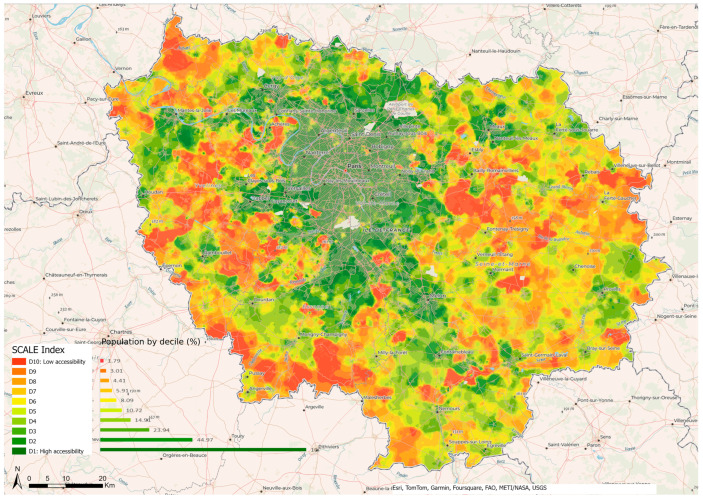
Accessibility to health care in Ile de France.

In Ile-de-France, i.e., the region containing Paris and with a high population density, accessibility is unequal between the different towns. Inner Paris has high accessibility with the highest deciles (D1 to D3) (Figure 3). However, there are also some other outlying areas of high accessibility that are not contiguous with this inner group. This second set comprises the municipalities situated to the north of an axis that takes in Aubervilliers, La Courneuve, and Saint-Denis. Other areas of high accessibility include the following: the east and northeast of Paris beyond the 20th arrondissement towards Montreuil, Bondy, Sevran, and Villepinte; further south towards Maisons-Alfort, Saint Maur-des-Fossés, as far as Sucy-en-Brie; and quite a large area to the southwest and west of Paris, stretching from Villejuif to Antony, Meudon, Boulogne-Billancourt, Nanterre, Neuilly-sur-Seine, and Levallois Perret. This area is also characterized by the presence of forests and woods, such as the Bois de Boulogne, the national forest of Meudon, and towns such as Rueil-Malmaison.

Except for these areas, the rest of Ile de France is characterized by degraded to poor accessibility, particularly in eastern Paris. This is the reflection of the well-known social demographics of the Île-de-France region, with its clear-cut delimitation between forested and industrial areas, and its large social inequalities between Paris and its outer suburbs, particularly the north-east, east and southeast. The latter areas are largely inhabited by populations that are poorer overall and who have less geographic mobility due to the weakness of the public transport network serving the districts and cities of the greater suburbs to the east of Paris. While these social factors influence access to employment, they also have an effect on accessibility to health facilities, as highlighted by the SCALE index in the field of health.

Maps for all regions are available in Supplementary Data (Figure S13). 

**Figure 4:**
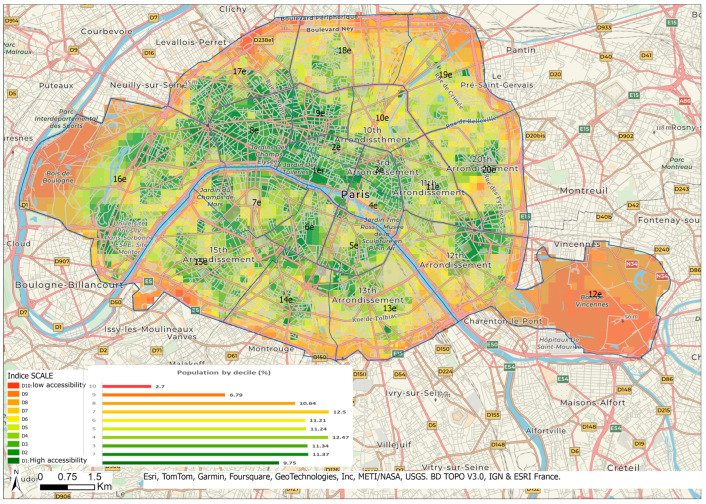
Accessibility to health care in Paris.

In the whole Ile-de-France area, the center of Paris has high scores, suggesting good accessibility to health care services in these areas (Figure 3). On a more detailed scale (Figure 4), disparities appear with the highest accessibility for residential areas located in the first, second, third, fourth, eighth, and ninth arrondissements and the lowest in the thirteenth, fourteenth, fifteenth, sixteenth, eighteenth, nineteenth, and twentieth.

To understand these discrepancies in Paris, supply and demand were considered separately (Figures S1–S13).

**Figure 5:**
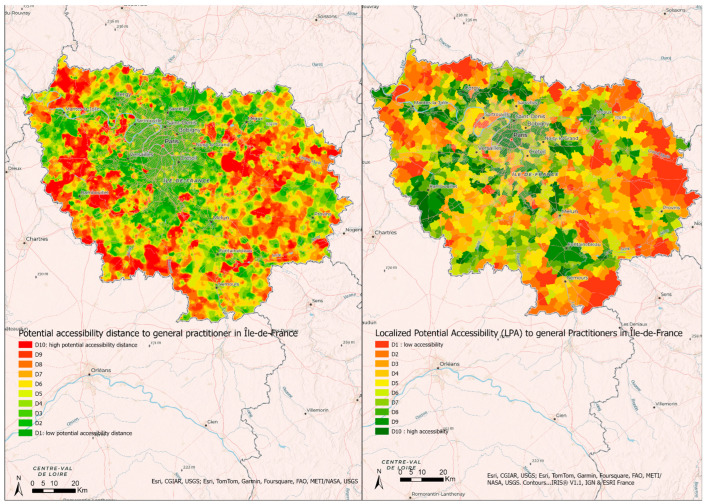
Comparison between potential accessibility distance and Localized Potential Accessibility to general practitioners (number of consultations per year per inhabitant) in Île-de-France.

Since the SCALE index is composite, each separate component may be used to identify specific features of accessibility to care (Figure 5). 

### 3.1. Comparison between Localized Potential Accessibility 2018 and the Potential Accessibility Distance to General Practitioners

The only index measuring accessibility in Metropolitan France is the Localized Potential Accessibility, which is available only at the municipal level. As it is calculated separately for different types of health professionals, it was not possible to compare it with the SCALE index, so we used the Potential Accessibility Distance, i.e., one of the components of the SCALE index (Figure 5).

Localized Potential Accessibility 2018 at municipal level (data available at URL: observatoire-des-territoires.gouv.fr accessed on 17 March 2023) demonstrates high accessibility to health care in Paris, Meaux, Saint Denis, Mantes la Jolie, Saint Maur des Fossés, Rambouillet, Evry, and Fontainebleau, and poor accessibility in the southwest and the east (Figure 5). The Potential Accessibility Distance was the greatest to the north of Mantes-la-Jolie, the north and the east of Rambouillet, and the south and to the east of Ile-de-France.

### 3.2. SCALE INDEX at Different Geographical Levels

By using the IRIS, it is possible to obtain different versions according to the aggregation criteria selected, i.e., the minimum, maximum, mean, or weighted mean per population. 

The distribution was as follows (Table 1):

The largest differences in ranking were obtained between the version using the minimum and the other versions (between 36% and 44% of identical rankings only). The version using the maximum compared to the two versions based on the average gave between 59% and 65% of IRIS classified identically. As expected, the greatest similarities in ranking were obtained between the version using the mean and the one using the weighted average with 77% of IRIS classified identically. 

The different versions of the index at the municipal level are shown in Table 1.

At the municipal level, the differences in rankings were more marked with only 23% and 31% of identical rankings between the minimum version and the other versions, and 42% and 49% between the maximum version and the average versions. Sixty-nine percent (69%) of municipalities were classified identically between the version based on a simple average and that using a population-weighted average (results not shown).

## 4. Discussion

In Metropolitan France, accessibility to health care is lower over an entire Y-shaped area stretching from the Normandy region, passing through Orléans, to the east around Troyes and then to the south towards the Massif Central. Accessibility is also poor in Corsica, in the rural regions of Nouvelle-Aquitaine, and in the Provence/Alpes/Cote d’Azur region. In Ile-de-France, accessibility is very unequal with inner Paris benefitting from high accessibility. 

These findings demonstrate that accessibility is a relative measure defined according to the accessibility of the other spatial units. The thresholds for categorizing accessibility, e.g., the 10% least accessible, depend on the distribution of the SCALE index for the geographical area under consideration. This can lead to different interpretations of the index. For example, a residential area may be considered as having low accessibility compared to other residential areas in the same region, but high accessibility compared to other areas in Metropolitan France.

The SCALE Index takes into consideration both accessibility and availability using floating sectors that can be obtained thanks to travel time to the nearest facilities. The geographical units that are usually used to evaluate accessibility are the municipality or the census tract, which are useful due to the huge amount of data available at these levels [28]. The resulting assumption is that accessibility is homogeneous for the entire spatial unit; yet, this is clearly not the case for both urban and rural areas since they do not reflect the real living environment. On the other hand, by using residential areas, it is possible to highlight heterogeneity in spatial units. It also allows accessibility to be evaluated in different geographical units from the largest to the smallest across Metropolitan France. Considering access to care in terms of access to a single professional is not in accordance with the population’s needs. Care can be administered in various forms by various professionals. This fact has to be taken into account when evaluating accessibility to health services. By combining the potential accessibility distance in a unique measure of accessibility, the SCALE index can serve as a composite index of accessibility to primary care. However, it is possible to consider all services independently by using the potential accessibility distance, since it can help in understanding the results obtained with the SCALE index. Low accessibility could be due to an accumulation of poor access to all professionals or very poor access to one of the professionals and average access to the others. The accessibility to care for the people living in these areas may, therefore, differ considerably, so policymakers and deciders would benefit from having this information upstream. Summary measures of accessibility to different health professionals have already been proposed [29] but never for the whole of France and for such a large panel of professionals (Table 2).

The SCALE index has much-added value thanks to its inclusion of the road network. Obviously, travelling 40 km in a car is not the same depending on whether the trip is made in the plain or the mountains, on small country roads or on a motorway. Travel time is also dependent on the time of day, a factor that was not taken into account. A sensitivity analysis in which these parameters are considered in calculating the index would make it possible to determine their impact. This impact might be particularly significant in urban areas where traffic may be dense during rush hours. 

The SCALE index has some limitations. First, it considers only travel time in cars, which is obviously a limitation, especially in urban areas where there are several modes of transport [29]. Second, even if the availability of care is taken into account, measuring it by the number of professionals is only a proxy. In fact, the professional’s practice (full-time or part-time) can affect it. Information on the professional and their speciality can also affect it, as can their reputation and whether they work in private practice, alone, or in a medical center with other health professionals. Their hours of work can also have an impact on the availability of health care. While information such as this is important, it could not be taken into account due to the absence of data at the level of this geographical unit. Third, only travel between home and health professionals’ practice was taken into account; yet, travel between workplace and practice might be more appropriate for working people [30]. 

As previously explained, the SCALE index can be calculated at all geographical scales. Nevertheless, since it is based on the residential area, it is preferable to use it at this scale, because a change of scale would imply an a priori hypothesis for the choice of the aggregation method. The aggregation criterion should be flexible and should be adapted to the objectives of the investigation. For example, if the focus is low accessibility to health facilities, the aggregation criterion using the maximum would be used.

## 5. Conclusions

Since accessibility is a multidimensional notion [31], the SCALE index could be computed for fields other than health and would, thus, be complementary to other sources of information. It would provide a multiscalar health accessibility index by considering a wide range of domains, such as education, culture, and justice in order to obtain a synthetic index of accessibility to all public facilities. Indeed, accessibility is a complex issue involving various factors that can be added and combined to assess their impact on different populations. The accumulation of inequalities of access to public services highlights the various facets of the social inequalities that affect different spatial units. Finally, we believe that the SCALE index could be used across Europe in the coming years. 

## Figures and Tables

**Table 1:** Distribution of SCALE index by census tract and by municipality according to different aggregation criteria.

	Aggregation Criteria for Each Spatial Unit							
	IRIS				Commune			
	Min	Max	Mean	Weighted Mean	Min	Max	Mean	Weighted Mean
Min	−21.9	−20.75	−20.75	−21.18	−21.9	−15.74	−17.18	−20.07
Mean	−5.18	−1.75	−3.03	−3.01	−3.11	1.15	−0.45	−0.56
Std	4.96	6.14	5.73	5.82	3.7	3.69	3.67	4
P25	−8.9	−5.47	−6.86	−6.98	−5.09	−1.04	−2.56	−2.85
P50	−4.15	−0.31	−1.75	−1.81	−2.58	1.1	−0.33	−0.31
P75	−1.31	2.43	0.97	1.07	−0.57	3.25	1.73	1.84
Max	18.1	25.5	23.3	25	18.06	25.45	23.29	24.96

**Table 2:** Comparison of methods used to measure accessibility.

Type	Density	Distance	2SFCA	APL	SCALe Index
			Density	Density	Distance
Geographic scale	Depends on application		Municipality n ≈ 36,000 or IRIS n ≈ 50,000	Municipality n ≈ 36,000	Residential area n ≈ 2.7 M, IRIS n ≈ 50,000, municipality n ≈ 36,000
Availability of caregivers	No	No	Number of professionals	Full-time equivalent number of caregivers	Pressure rate estimated by Voronoï mosaic
Strength	Easy to compute	Intuitive for the public	Availability is taken into account Floating catchment	Availability is taken into account using full-time equivalent Floating catchment	No administrative boundary Multiscalar Include various type of primary caregivers Availability is taken into account Floating catchment
Weakness	Administrative boundary Unique values for each geographical unit No floating catchment area Only based on spatial accessibility	Only one facility Does not take into account availability Only based on spatial accessibility	Administrative boundary Only based on spatial accessibility	Uniscalar Administrative boundary Only based on spatial accessibility	Quality of geolocalization of primary caregivers Only based on spatial accessibility Includes various types of primary care givers Multiscalar

## Data Availability

Some of the data on which this study is based are freely available, and some are legally restricted by third-party owners. The SCALE index (in shp format) has been deposited in the repository named data.gouv.fr (Open platform for French public data) and can be accessed freely via Accessibilité aux soins de premier recours en France métropolitaine—data.gouv.fr. The Permanent Facilities Database (BPE) 2013 and Census 2010 databases are the property of INSEE who provide them only on request. Requests may be sent to: https://www.insee.fr/fr/information/1912226 (accessed on 3 June 2013). More recent BPE databases are freely available from INSEE online via https://www.insee.fr/fr/statistiques/3568638?sommaire=3568656 (accessed on 15 November 2023). Contours… IRIS data belong to the Institut Geographique National (IGN) and has been replaced by IRIS… GE, requests may be sent to https://www.insee.fr/fr/information/1912226 (accessed on 13 June 2023). IRIS… GE are freely available at https://geoservices.ign.fr/irisge (accessed on 13 June 2023). BD TOPO data are also the property of IGN and may be freely accessed via https://geoservices.ign.fr/bdtopo (accessed on 13 June 2023). Navstreets data belong to HERE^®^ and are not freely available.

## Supplementary Materials

The following supporting information can be downloaded at: https://www.mdpi.com/article/10.3390/ijerph21030276/s1. Figure S1: SCALE Index in Bourgogne-Franche-Comté; Figure S2: SCALE Index in Grand-Est; Figure S3: SCALE Index in Normandie; Figure S4: SCALE Index in Bretagne; Figure S5: SCALE Index in Centre-Val de Loire; Figure S6: SCALE Index in Corse; Figure S7: SCALE Index in Hauts-de-France; Figure S8: SCALE Index in Nouvelle-Aquitaine; Figure S9: SCALE Index in Occitanie; Figure S10: SCALE Index in Provence-Alpes-Côte d’Azur; Figure S11: SCALE Index in Pays-de-la-Loire; Figure S12: SCALE Index in Auvergne-Rhône Alpes; Figure S13: Population density and number of primary healthcare places in Paris.

## Abbreviations

APL: localized potential accessibility; 2SFCA: Two-Step Floating Catchment Area; E2SFCA: Enhanced two-step floating catchment area; 3SFCA: a three-step floating catchment area; IRIS: Ilots Regroupés pour l’Information Statistique; SCALE: Spatial aCessibility multiscALar.
