# Supplementary figures and images for: Spatial Accessibility to Primary Care in Metropolitan France: Results Using the SCALE Spatial Accessibility Index for All Regions
Source: Int J Environ Res Public Health. 2024 Feb 28;21(3):276. doi: 10.3390/ijerph21030276 (PMC10970661; doi:10.3390/ijerph21030276)

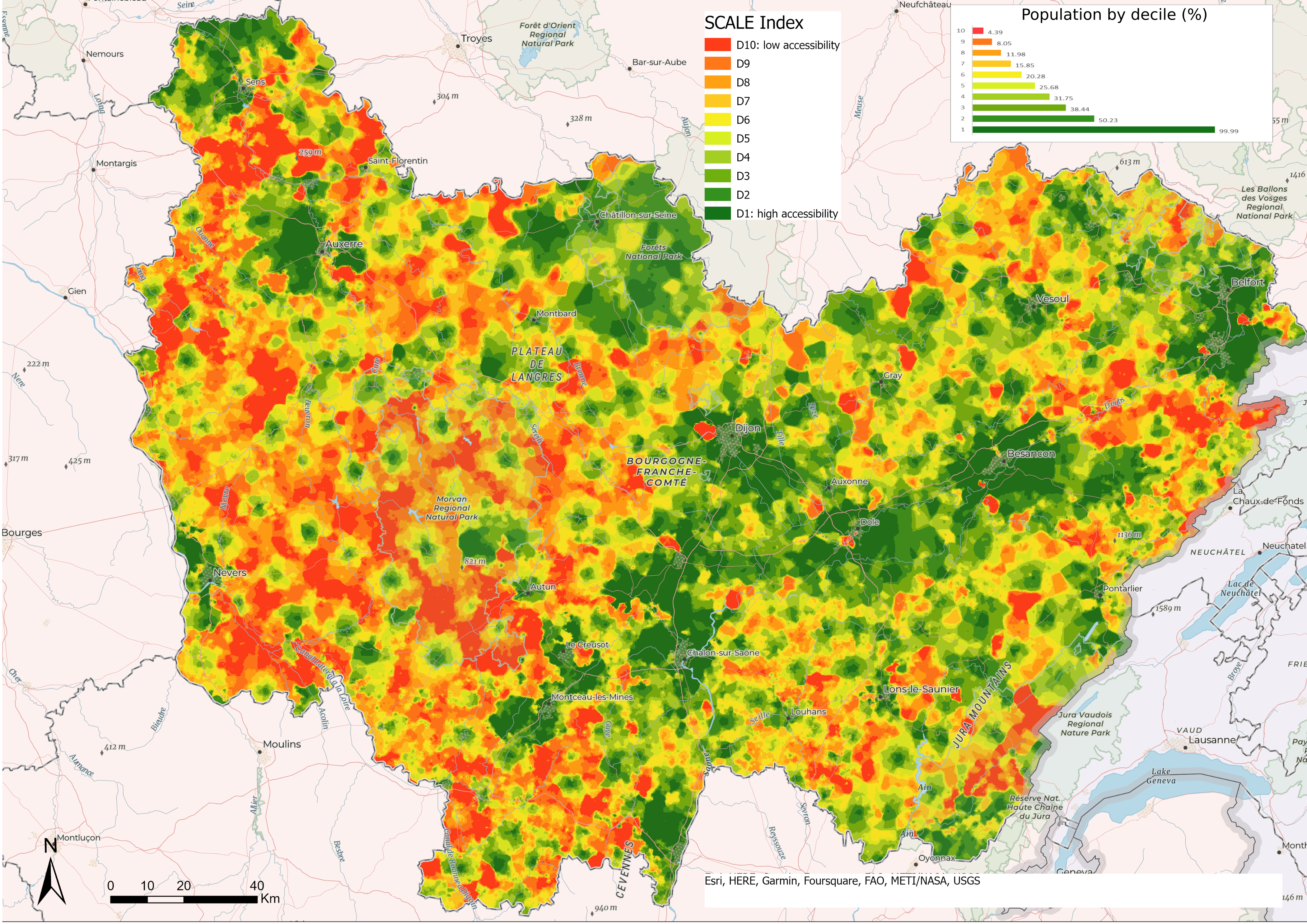

Supplement: Supplementary file 1 [file ijerph-21-00276-s001.zip › supplementary_files/S1 Figure.png]

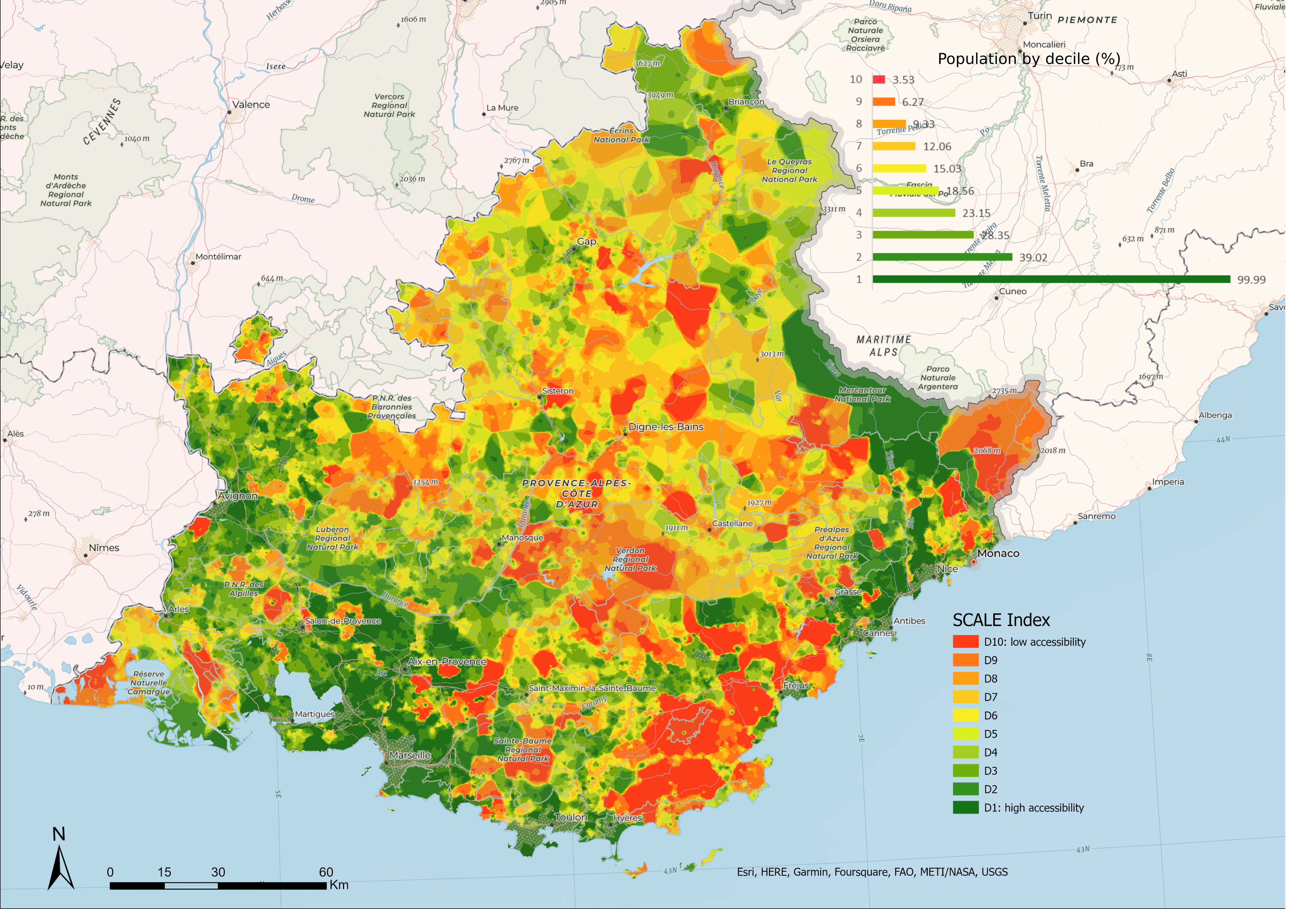

Supplement: Supplementary file 1 [file ijerph-21-00276-s001.zip › supplementary_files/S10 Figure.png]

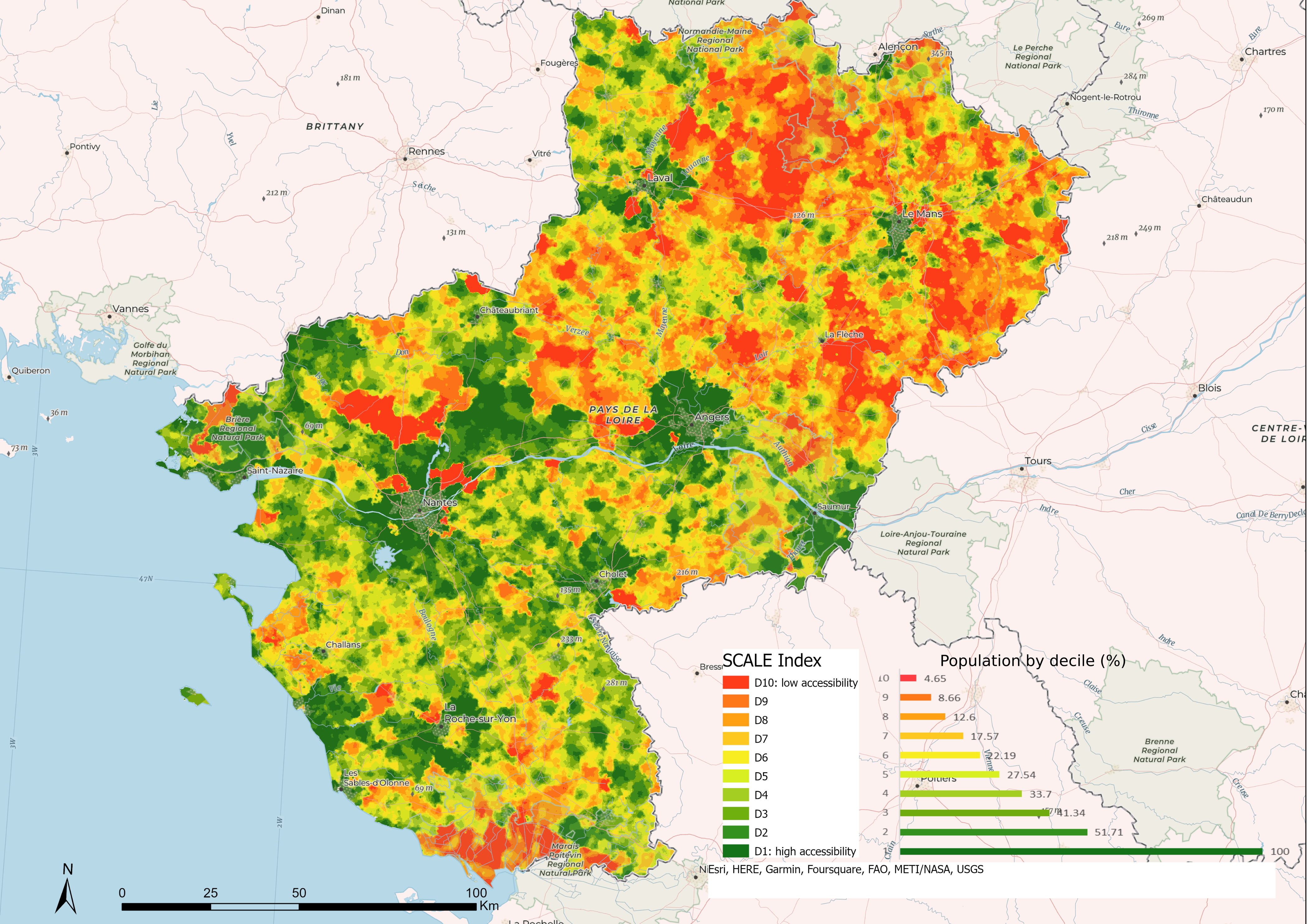

Supplement: Supplementary file 1 [file ijerph-21-00276-s001.zip › supplementary_files/S11 Figure.png]

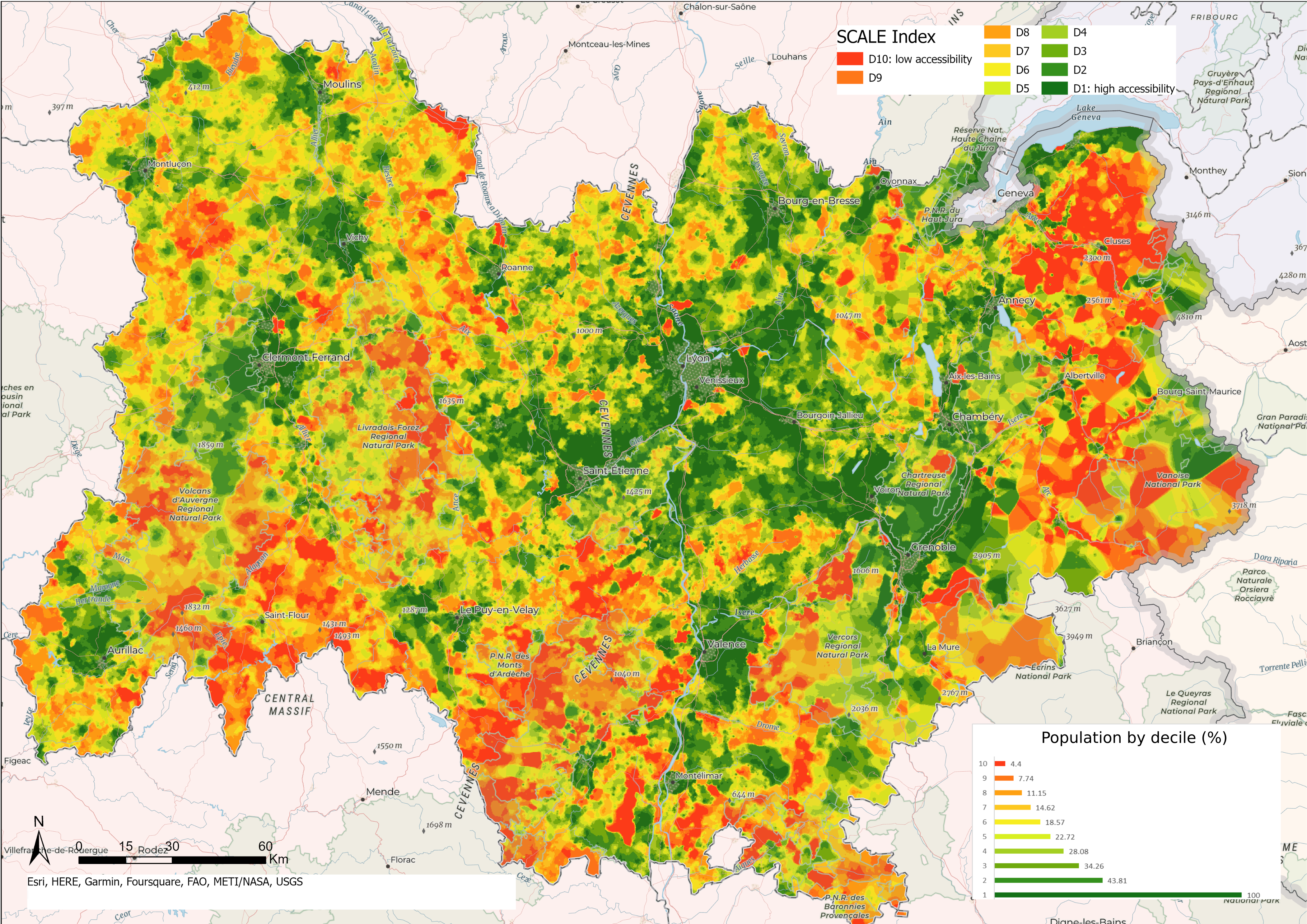

Supplement: Supplementary file 1 [file ijerph-21-00276-s001.zip › supplementary_files/S12 Figure.png]

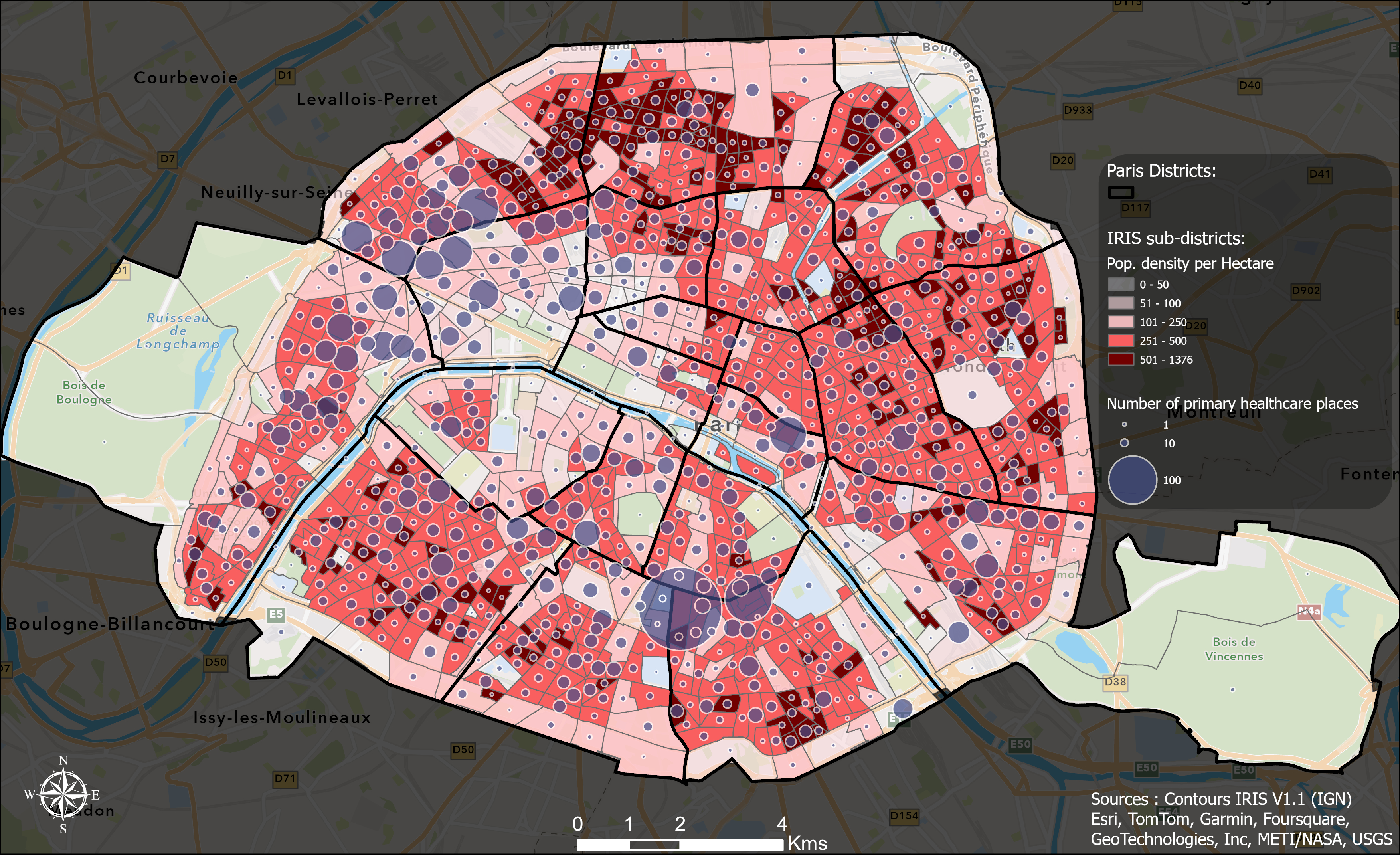

Supplement: Supplementary file 1 [file ijerph-21-00276-s001.zip › supplementary_files/S13 Figure.jpg]

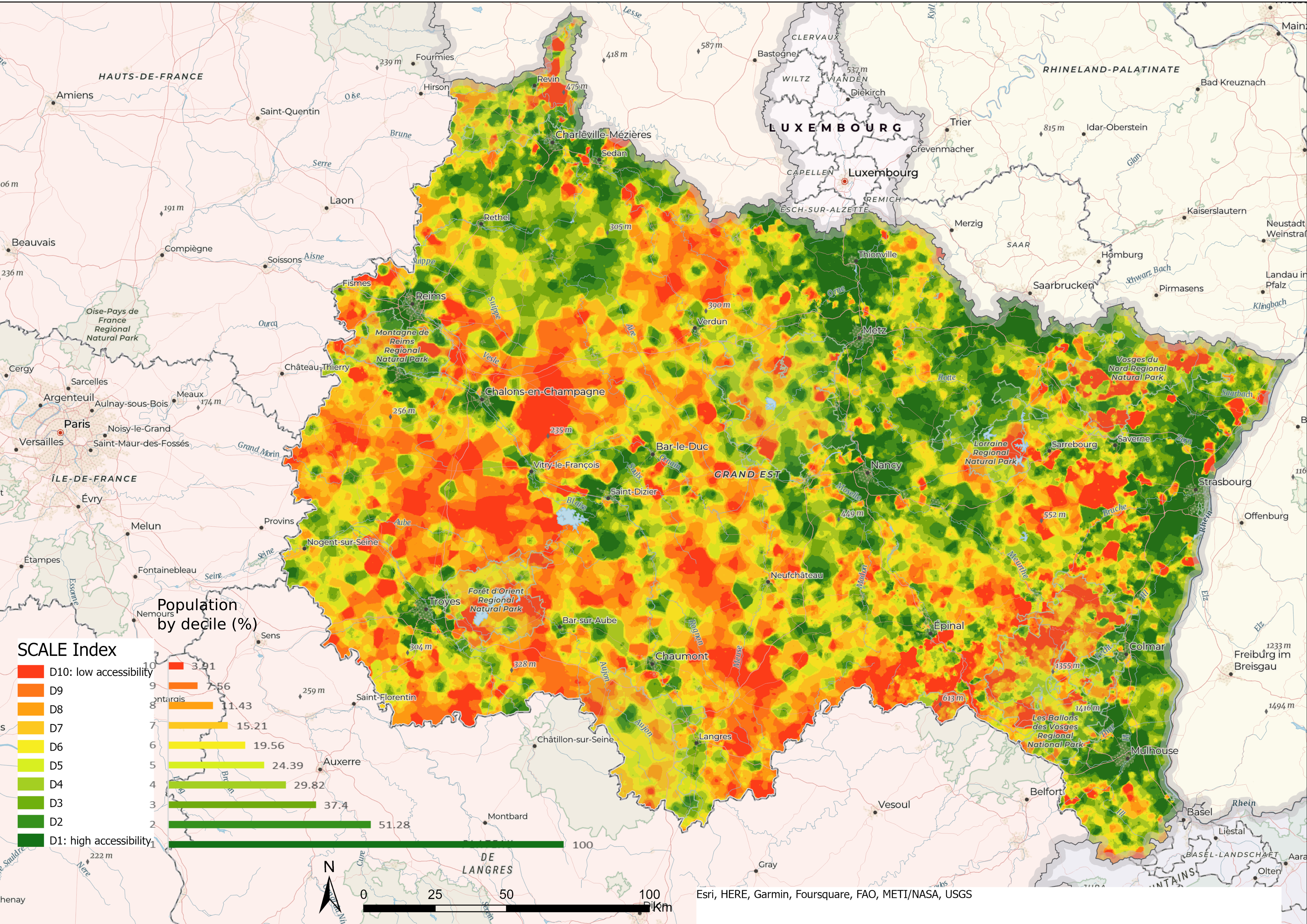

Supplement: Supplementary file 1 [file ijerph-21-00276-s001.zip › supplementary_files/S2 Figure.png]

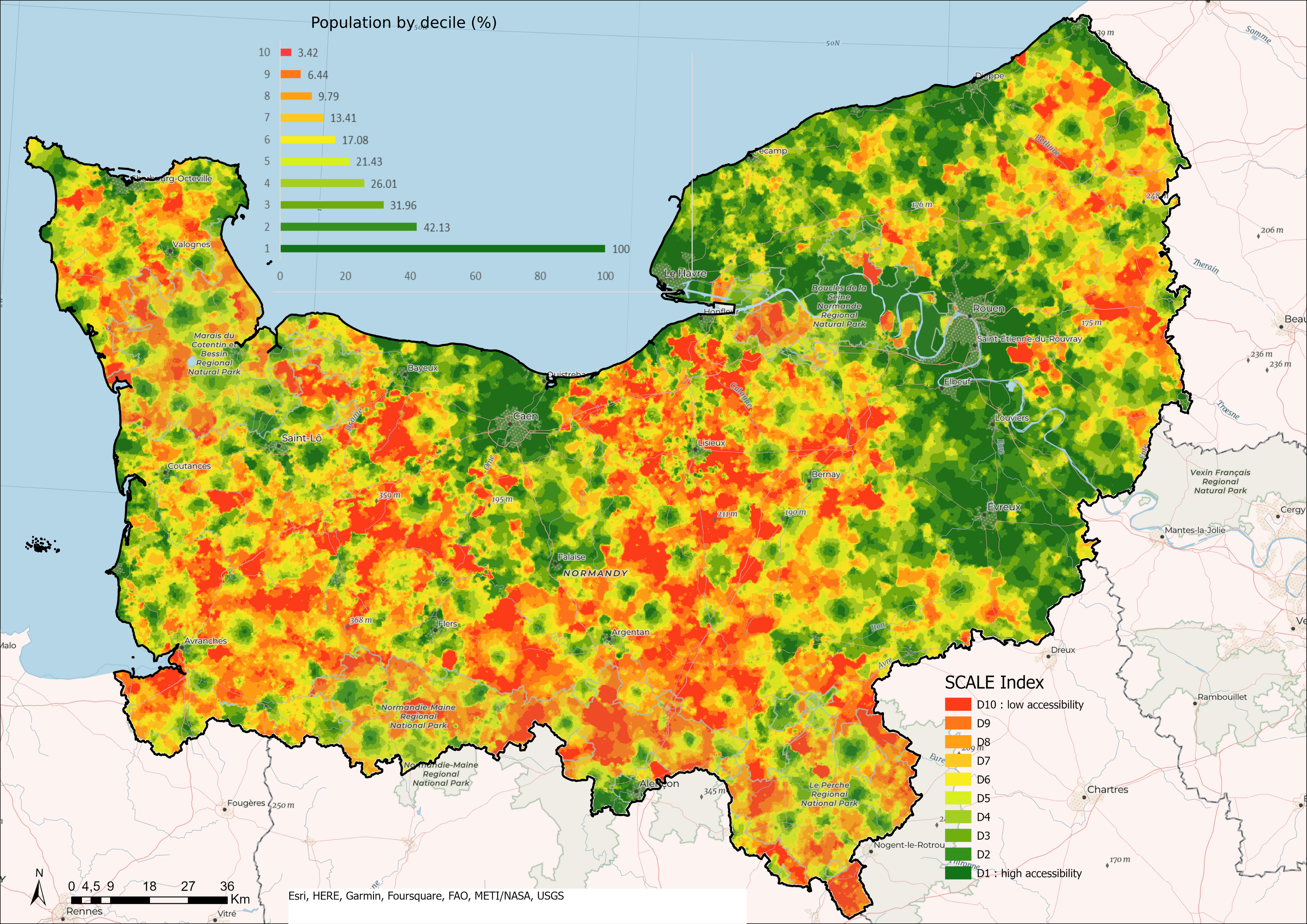

Supplement: Supplementary file 1 [file ijerph-21-00276-s001.zip › supplementary_files/S3 Figure.png]

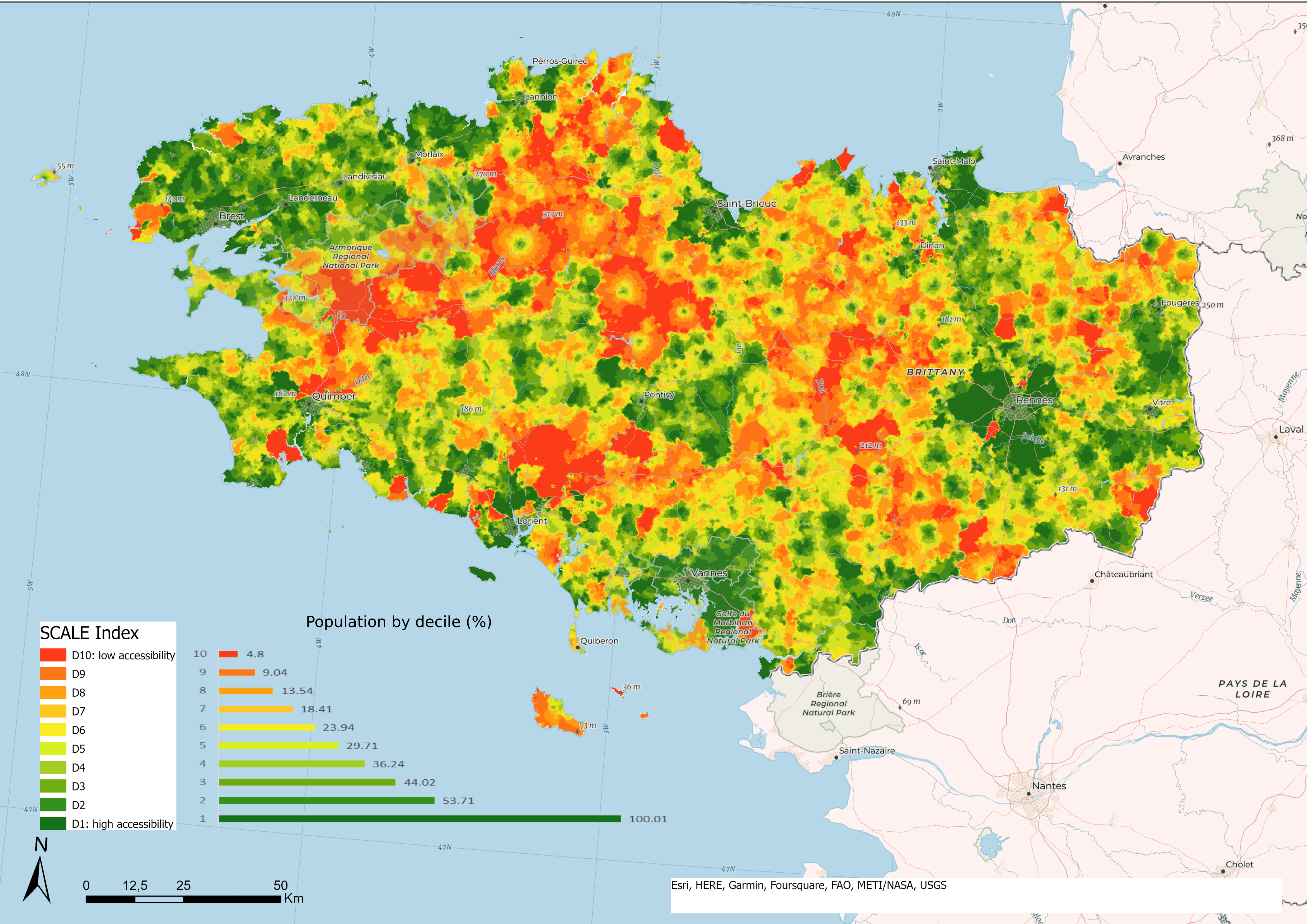

Supplement: Supplementary file 1 [file ijerph-21-00276-s001.zip › supplementary_files/S4 Figure.png]

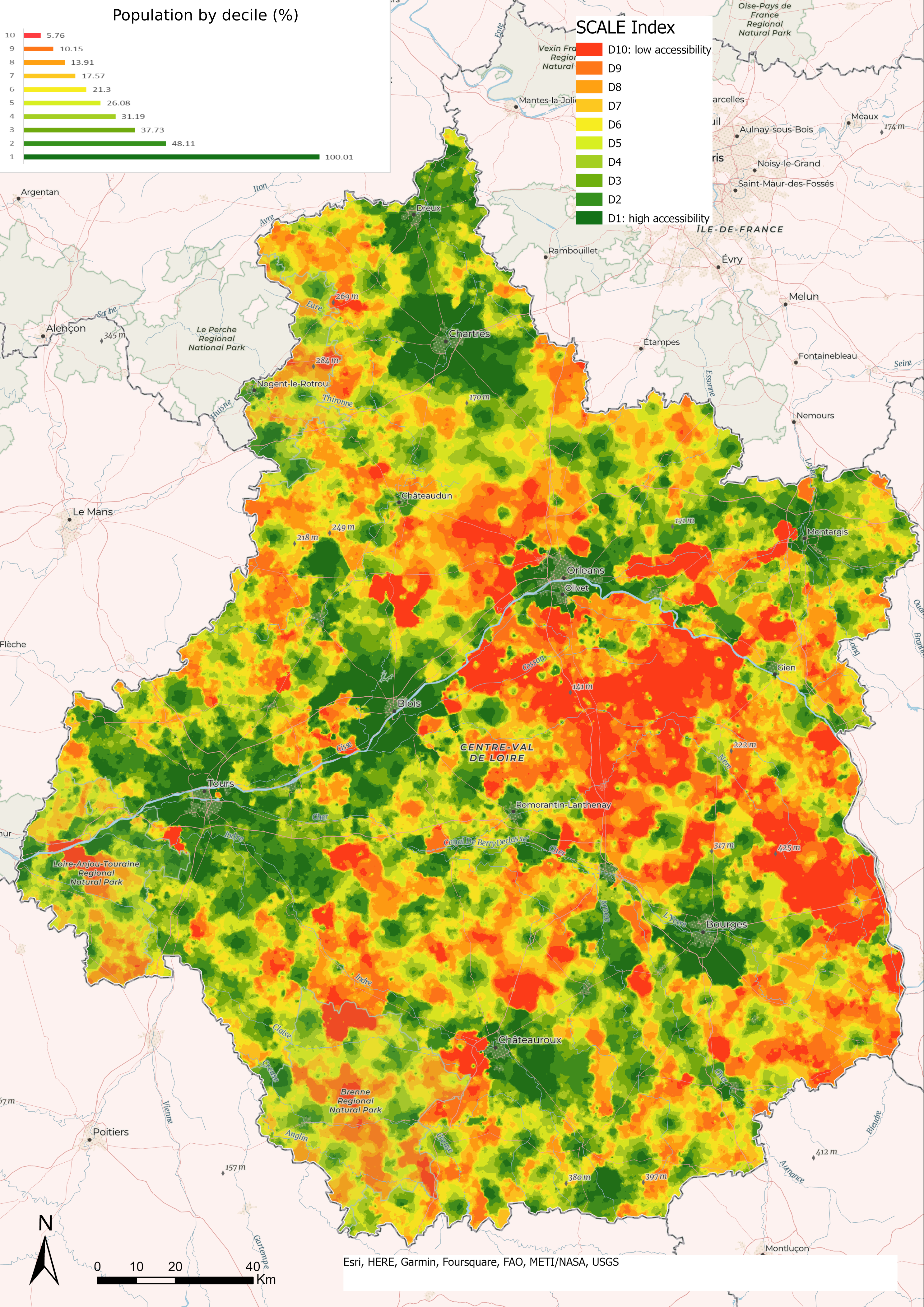

Supplement: Supplementary file 1 [file ijerph-21-00276-s001.zip › supplementary_files/S5 Figure.png]

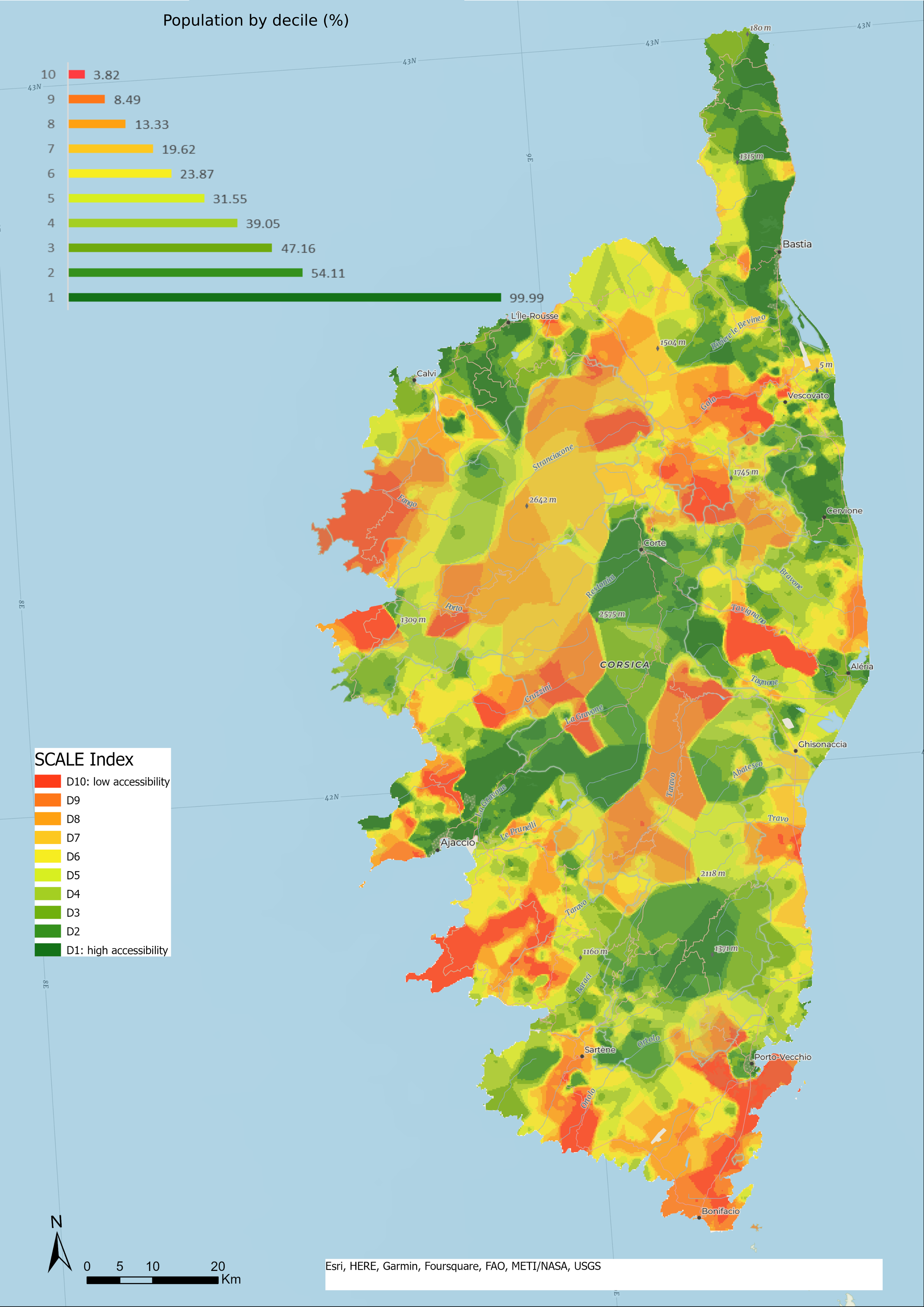

Supplement: Supplementary file 1 [file ijerph-21-00276-s001.zip › supplementary_files/S6 Figure.png]

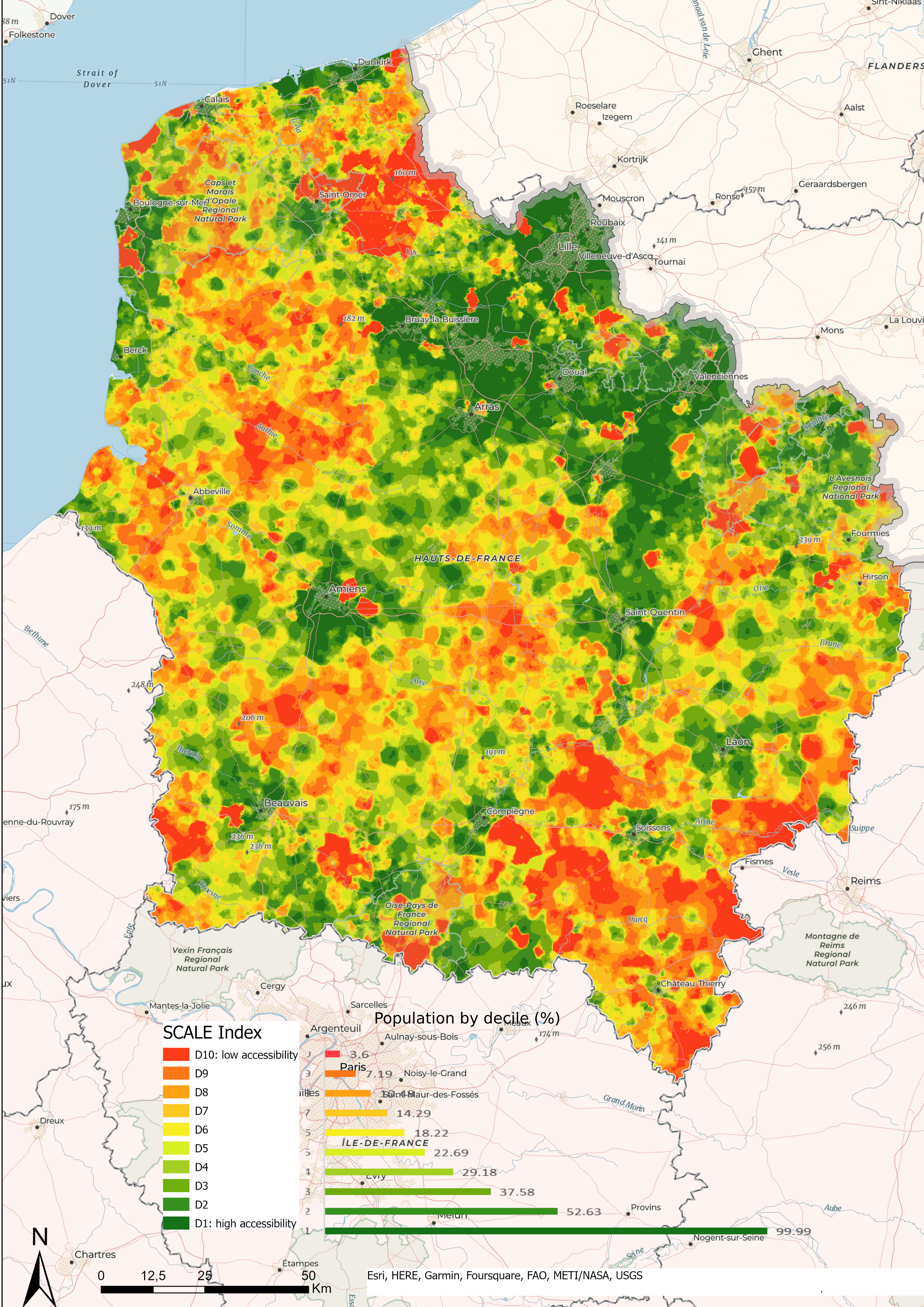

Supplement: Supplementary file 1 [file ijerph-21-00276-s001.zip › supplementary_files/S7 Figure.png]

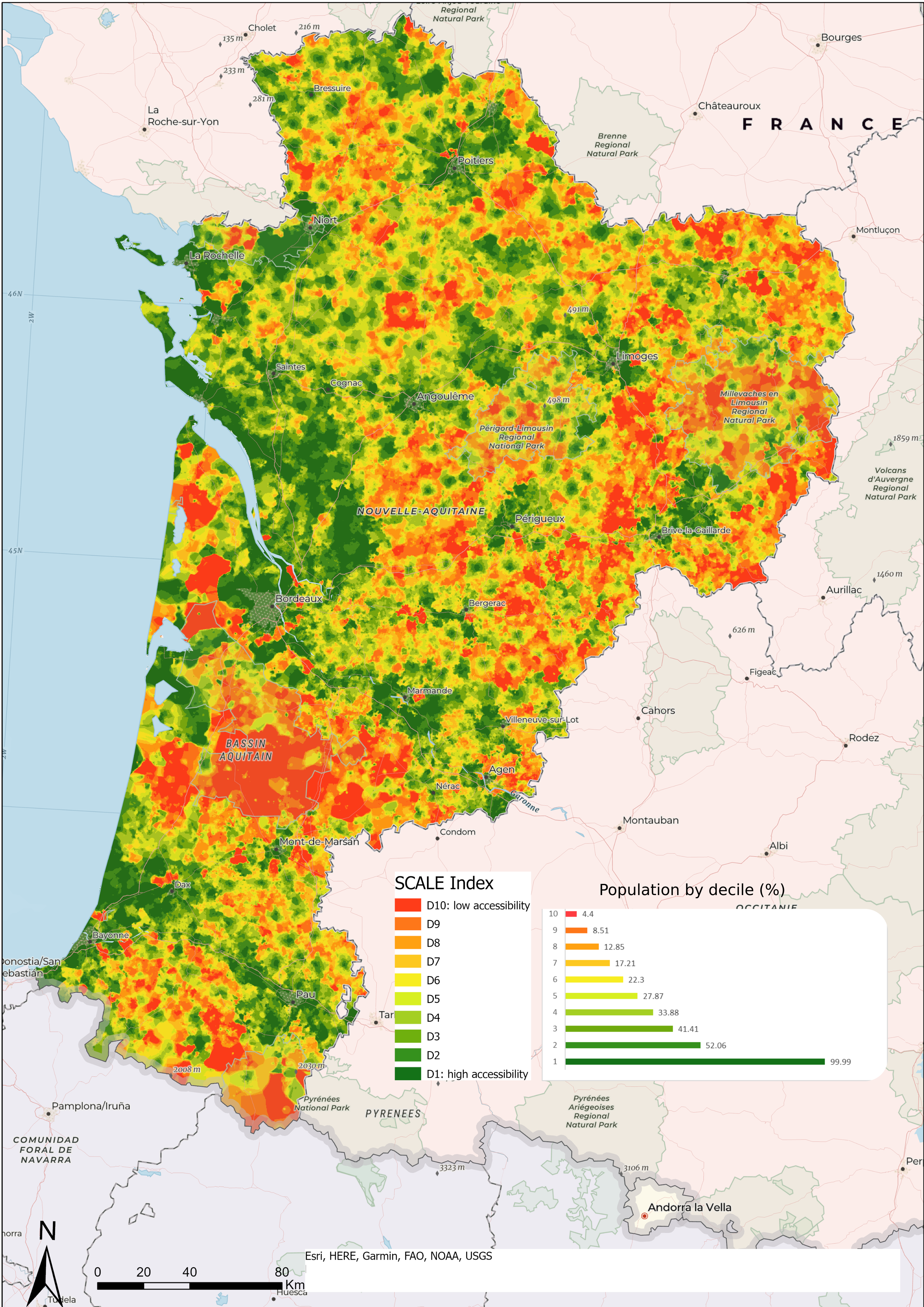

Supplement: Supplementary file 1 [file ijerph-21-00276-s001.zip › supplementary_files/S8 Figure.png]

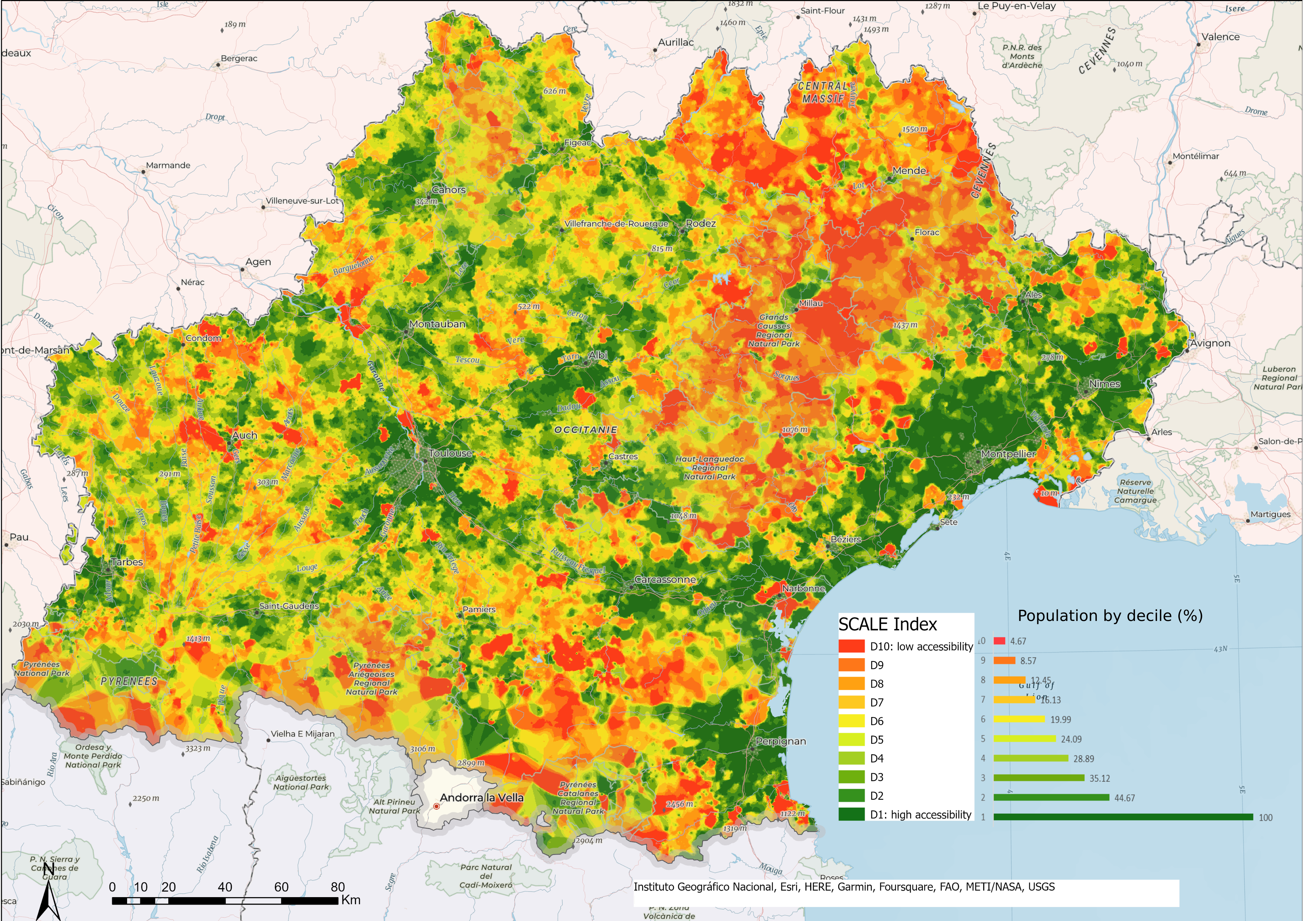

Supplement: Supplementary file 1 [file ijerph-21-00276-s001.zip › supplementary_files/S9 Figure.png]
